# Reproducibility of autonomic cardiovascular function and hemodynamics at rest and during recovery from exercise

**DOI:** 10.14814/phy2.70007

**Published:** 2024-08-18

**Authors:** Venla P. Ylinen, Rasmus I. P. Valtonen, M. Juhani Junttila, Risto Kerkelä, Heikki V. Huikuri, Mikko P. Tulppo

**Affiliations:** ^1^ Research Unit of Biomedicine and Internal Medicine University of Oulu Oulu Finland; ^2^ Biocenter Oulu University of Oulu Oulu Finland; ^3^ Medical Research Center Oulu Oulu University Hospital and University of Oulu Oulu Finland

**Keywords:** aerobic exercise, autonomic nervous system, blood pressure, heart rate variability

## Abstract

Smartwatches and home‐based blood pressure (BP) devices have permitted easy use of heart rate variability (HRV) and BP to identify the recovery status of users after acute exercise training. The reproducibility of HRV and BP after exercise in healthy young participants is not well known. Eighteen participants (age 27 ± 6 years, female *n* = 8) performed test and retest aerobic exercises (cycling, 30 min, 60% of peak workload, W) and a control session in randomized order. RMSSD, high and low‐frequency power of RR intervals, and BP were measured at rest and 30–60 min after interventions. The relative reproducibility was assessed by the intraclass correlation coefficient (ICC) and 95% confidence interval (95% CI). The absolute reproducibility was evaluated using the coefficient of variation (CV%). HRV indices revealed moderate‐to‐excellent reproducibility at rest (ICC 0.81–0.86; 95% CI 0.53–0.95) but not after exercise (ICC −0.06 to 0.60; 95% CI −1.85 to 0.85). Systolic BP had a good‐to‐excellent reproducibility before (ICC 0.93; 95% CI 0.81–0.98, CV% 4.2) and after exercise (ICC 0.93; 95% CI 0.81–0.97, CV% 4.2). The reproducibility of HRV indices is poor after exercise in young participants. However, the reproducibility of BP is excellent at rest and after aerobic exercise.

## 1 INTRODUCTION

The measurement of cardiac autonomic function using heart rate variability (HRV) techniques has become popular among users of wearable devices such as heart rate monitors, smart rings, and smartwatches. These devices are very easy to use, and HRV data can be recorded and shown to the users automatically after normal daily or nighttime routines. One potential application is to identify the users' recovery status or “readiness” after an acute exercise training using short‐term HRV measurements (Kinnunen et al., 2020; Lamberts et al., 2024). For example, the Oura smart ring measures short‐term HRV values in 5‐min windows from which the average values are calculated (Kinnunen et al., 2020). Apple and Polar smartwatches (Nuuttila et al., 2022) and the Firstbeat Bodyguard System (Seipäjärvi et al., 2022) similarly measure short‐term HRV values and provide users with the average values from the measurement period. Home‐based blood pressure (BP) measurements have also become popular among middle age and older population (Springer et al., 2022). The reproducibility of HRV and BP measurements after an acute aerobic exercise, when the heart rate is returned to the baseline or near to the baseline level, is not well known.

Postexercise BP has become an interesting area in the scientific community during the last decade. This is partly due to the strong association between acute BP responses after exercise and BP responses after chronic exercise training. A large reduction of BP after a single aerobic exercise session predicts a large BP reduction after chronic aerobic exercise training at the individual level among patient populations (Kiviniemi et al., 2015; Liu et al., 2012). A short‐term BP is regulated by sympathetic and parasympathetic outflow to the heart and peripheral vasculature. These regulatory mechanisms can be estimated noninvasively by HRV, systolic BP variability, and spontaneous baroreflex sensitivity measurements (BRS). This study's primary aim was to identify the reproducibility of HRV indices, systolic BP, systolic BP variability, and spontaneous BRS before and after moderate‐intensity aerobic continuous exercises and control measurements in young, normotensive participants without any medications. We hypothesized that reproducibility of HRV indices is weaker during recovery from exercise than at rest due to the interindividual differences in cardiac autonomic regulation particularly during recovery from exercise. Furthermore, we hypothesized that reproducibility of systolic BP is good at rest and during recovery from exercise as an outcome of all BP regulatory mechanisms.

## 2 MATERIALS AND METHODS

### 2.1 Participants

The participants were healthy males (*n* = 10) and females (*n* = 8) recruit by an advertisement among medical students and the local sport community (age 27 ± 6 years). The menstrual cycle was not controlled. The inclusion criteria were BP <130/85 mmHg for 1 week of home monitoring, body mass index (BMI) <30 kg/m^2^, age 20–40 years, no medication and no smoking or use of any other nicotine products. The Northern Ostrobothnia Hospital District's Ethical Committee approved this study. The volunteer participants signed a written informed consent form for the study and had the right to terminate the study at any time. The participants were informed not to exercise 48 h before every visit and to avoid caffeine (4 h before tests) and alcohol (48 h before tests).

### 2.2 Preliminary evaluation

The participants underwent anthropometric measurements on the first visit prior to the maximal bicycle ergometer test (Monark, Sweden). The visit included resting BP measurements that were treated as familiarizing them to the lab setting and BP measuring. The maximal test was started from 40 W and was increased by 15 W for women and 20 W for men every 2 min until exhaustion and gas exchange was monitored (Vyntus™ CPX, Vyaire Medical, Chicago, USA). BP measurements at home were performed after the first visit (Omron M6 Comfort, Omron healthcare Co., Japan) based on a protocol of three measurements taken at least 2 mins apart in the morning and evening for 7 days and always at the same time of day. Home BP was controlled, since we wanted to be sure that all the subjects were normotensive. Second, we wanted to compare home BP measurements and laboratory BP measurements to exclude potential subjects with white coat effect (none).

### 2.3 Experimental protocol

The participants performed two equal intensity, 30‐min aerobic exercises. The baseline BP (Schiller BP 200+, Switzerland) was measured from the left arm after 10 min of sitting in a quiet room before the exercises. The measurement was repeated triplicate (2 min between each) and average values were calculated. The cardiovascular autonomic function and hemodynamics were measured in a sitting position thereafter. The exercises were conducted in a temperature‐controlled laboratory (22°C) on a bicycle ergometer on separate days with at least 2 days (48 h) between the sessions. Both exercises were performed at intensities of 60% of an individual maximal load (W) at the same time of day to minimize the diurnal variation in cardiovascular variables. The BP was measured every 5 min during the 30‐min exercise. The HR was recorded continuously and the RPE was evaluated subjectively from 0 to 20 during the exercises. The participants remained seated in a laboratory during the recovery phase (60 min). The postexercise BP was measured in 5‐min intervals over the whole 60‐min recovery. The 30–35‐min and 55–60‐min postexercise BP was measured three times at 2‐min intervals and the average values were calculated. The control session without exercise was performed to investigate the time effect on cardiovascular variables.

### 2.4 Cardiovascular autonomic function

Standard lead II ECG (Cardiolife; Nihon Kohden, Tokyo, Japan), breathing frequency (PneumoTrace, ADInstruments, Australia), and BP by finger photoplethysmography (Nexfin; BMEYE Medical Systems, Amsterdam, the Netherlands) were recorded in the seated position. Signals were connected to the laptop with an analog‐to‐digital transformer with a 1000 Hz sampling frequency (Power Lab/8SP, ADInstruments, Australia) managed with Labchart software (v7.3.2, ADInstuments, Australia). These signals were recorded for 5 min at the baseline and 30–35‐min postexercise condition. The time series of RR‐interval and beat‐to‐beat systolic BP were extracted as described earlier (Perkiömäki et al., 2019). We then computed the root mean squared of successive differences (RMSSD) of RR intervals and spectral estimates for stabilized periods of baseline and postexercise conditions on LF (0.04–0.15 Hz) and HF (0.15–0.4 Hz) bands of RR intervals and BP variability by applying FFT (Welch's method, sequence length 128, overlapping 50%). BRS from LF band was calculated as described earlier (Perkiömäki et al., 2019).

### 2.5 Hemodynamics

Stroke volume (SV), cardiac output (CO), and systemic vascular resistance (SVR) were evaluated non‐invasively from the left arm using the Mobil‐O‐Graph oscillometric device (I.E.M.‐GmbH, Germany) at the baseline and recovery phases (the latter was 45 min. after exercise).

### 2.6 Statistics

The variables were transformed into natural logarithms (LF, HF, BP‐LF, and BRS‐LF) in the case of skewed distribution. Analysis of variance for repeated measures, including control and exercise measurements, was used to investigate the exercise effects followed by Bonferroni post hoc test. The relative reproducibility was assessed using intraclass correlation coefficient (ICC) and is presented as ICC and 95% confidence interval (CI) recommend by Koo et al. (2016). The reproducibility was defined as poor if the 95% CI of ICC was ≤0.50, moderate between 0.51 and 0.75, good between 0.76 and 0.90, and excellent if ≥0.91 (Koo & Li, 2016). Absolute reproducibility was evaluated using the coefficient of variation (CV%) (Costa et al., 2016). Person's correlation analysis was performed to study the association between the change in BP and other variables. The analyses were performed by IBM SPSS 28.0 (IBM Corp., Armonk, NY, USA) and the *α* was set a priori at 0.05.

## 3 RESULTS

Characteristics of the participants are shown in Table 1. There were no differences in heart rate, RPE or BP between exercises I and II (Table 1). Table 2 shows the results of autonomic function and hemodynamics at the baseline and postexperiment conditions for the control measurements and exercises. The systolic BP decreased 60 min after exercise compared to the control measurement (time × group interaction *p* < 0.012) and was significantly lower in post hoc analysis after exercises (113 ± 13 and 113 ± 12 mmHg, *p* < 0.05 for both) compared to the control measurement (118 ± 12 mmHg).

**TABLE 1:** Characteristics of the study participants.

	Mean ± SD (*n* = 18)
Male, *n* (%)	10 (56)
Age, years	27 ± 6
Height, cm	175 ± 9
Weight, kg	7 ± 13
Boby mass index, kg/m^2^	23 ± 3
Body fat, %	16 ± 6
BP at home	
Systolic BP, mmHg	111 ± 11
Diastolic BP, mmHg	70 ± 7
Mean BP, mmHg	83 ± 8
Exercise capacity	
Heart rate max, bpm	192 ± 8
Workload max, watts	245 ± 53
VO_2_max, mL/kg/min	45 ± 7
RER max	1.11 ± 0.03
Exercise I	
Mean load, watts	147 ± 30
Heart rate, % of max	76 ± 5
Systolic BP, mmHg	169 ± 23
RPE, Borg's scale	12 ± 2
Exercise II	
Mean load, watts	147 ± 30
Heart rate, % of max	73 ± 5
Systolic BP, mmHg	169 ± 21
RPE, Borg's scale	12 ± 1

Abbreviations: BP, blood pressure; RER, respiratory exchange ratio; RPE, Rated Perceived Exertion Scale (0–20).

**TABLE 2:** Autonomic regulation and blood pressure at baseline and after control and exercise interventions.

	Control		Exercise I		Exercise II		Anova for repeated measures		
	Pre	Post	Pre	Post	Pre	Post	Time	Group	Interaction
Autonomic function 30–35 min post									
Breathing freq, Hz	0.24 ± 0.06	0.24 ± 0.06	0.25 ± 0.05	0.25 ± 0.06	0.25 ± 0.05	0.25 ± 0.06	0.678	0.601	0.703
Heart rate, bpm	68 ± 11	60 ± 9	68 ± 11	72 ± 10*	66 ± 11	70 ± 10*	0.719	0.155	<0.001
Sys BP, mmHg	122 ± 9	117 ± 12	124 ± 15	114 ± 12*	123 ± 12	114 ± 12*	<0.001	0.965	0.068
Dia BP, mmHg	74 ± 5	73 ± 7	73 ± 6	73 ± 8	72 ± 7	72 ± 8	0.449	0.858	0.756
Mean BP, mmHg	90 ± 6	86 ± 8	90 ± 7	86 ± 9	89 ± 8	86 ± 9	<0.001	0.896	0.781
RMSSD, ln ms	3.7 ± 0.9	3.9 ± 0.7	3.7 ± 0.7	3.5 ± 0.5*	3.8 ± 0.7	3.8 ± 0.6	0.766	0.540	0.021
HF‐power, ln ms^2^	6.51 ± 1.68	6.71 ± 1.36	6.41 ± 1.44	6.21 ± 1.02	6.62 ± 1.42	6.62 ± 1.15	0.971	0.719	0.449
LF‐power, ln ms^2^	6.69 ± 1.43	7.02 ± 1.32	6.91 ± 1.11	6.93 ± 0.82	6.75 ± 1.10	7.25 ± 1.02	0.038	0.923	0.328
BP‐LF, ln mmHg^2^	1.88 ± 1.01	1.97 ± 0.72	1.95 ± 1.10	2.40 ± 0.66	1.88 ± 0.76	2.59 ± 0.73	<0.001	0.421	0.085
BRS‐LF, ln ms/mmHg	2.38 ± 0.65	2.58 ± 0.58	2.54 ± 0.55	2.31 ± 0.39	2.47 ± 0.62	2.34 ± 0.48	0.416	0.879	0.020
Hemodynamics 40–45 min post									
Stroke volume, mL	81 ± 19	91 ± 19	77 ± 15	78 ± 16*	89 ± 16**	82 ± 22	0.658	0.192	0.041
Cardiac output, L/min	5.16 ± 0.66	5.08 ± 0.71	4.84 ± 0.66	5.32 ± 0.76	5.34 ± 0.59	5.24 ± 0.96	0.455	0.479	0.129
SVR, dyn·s·cm^−5^	1484 ± 182	1485 ± 258	1576 ± 190	1375 ± 158	1400 ± 177	1391 ± 222	0.070	0.136	0.056
Blood pressure 55–60 min post									
Heart rate, bpm	66 ± 11	57 ± 9	65 ± 11	64 ± 10*	64 ± 11	62 ± 10*	<0.001	0.593	0.006
Sys BP, mmHg	122 ± 9	118 ± 12	124 ± 15	113 ± 13*	123 ± 12	113 ± 12*	<0.001	0.840	0.012
Dia BP, mmHg	74 ± 5	75 ± 7	73 ± 6	70 ± 7	72 ± 7	71 ± 8	0.172	0.316	0.318
Mean BP, mmHg	90 ± 6	89 ± 8	90 ± 7	85 ± 8	89 ± 8	85 ± 9	<0.001	0.487	0.068

Abbreviations: BP, blood pressure; BP‐LF, low frequency power of beat‐to‐beat systolic blood pressure; BRS‐LF, baroreflex sensitivity from low frequency band; HF, high frequency power of RR intervals; LF, low frequency power of RR intervals; RMSSD, root mean square of successive differences between RR inetervals; SVR, systemic vascular resistance.

* Post hoc test *p* < 0.05 between exercise and control post exercise values.

** *p* < 0.05 between exercises I and II at rest.

Table 3 shows the relative and absolute reproducibility variables before and after exercises. HRV indices revealed moderate‐to‐excellent relative reproducibility at rest (95% CI; 0.53–0.95) but not after exercise (95% CI; −1.85 to 0.85). BRS, SV, CO, and SVR had poor‐to‐good relative reproducibility before (95% CI; −0.72 to 0.89) and after exercise (95% CI −0.72 to 0.89). Systolic BP had a good‐to‐excellent relative reproducibility before (95% CI; 0.81–0.98) and after exercise (95% CI; 0.81–0.97). The absolute reproducibility was the best for systolic BP at rest and in recovery (CV% 4.2 for both).

**TABLE 3:** Reproducibility of different variables at baseline before and after exercises (*n* = 18).

	Baseline			Recovery		
	ICC	95% CI	CV%	ICC	95% CI	CV%
Autonomic function 30–35 min post						
Heart rate, bpm	0.832	0.561–0.937	8.7	0.863	0.638–0.948	6.5
Sys BP, mmHg	0.926	0.810–0.975	4.2	0.942	0.844–0.978	3.6
Dia BP, mmHg	0.862	0.632–0.948	4.7	0.712	0.214–0.893	7.7
Mean BP, mmHg	0.894	0.718–0.960	3.8	0.867	0.642–0.950	4.9
RMSSD, ln ms	0.810	0.538–0.919	9.1	0.306	−0.459‐0.693	12.4
HF‐power, ln ms^2^	0.864	0.643–0.949	10.8	0.603	0.002–0.848	12.6
LF‐power, ln ms^2^	0.841	0.581–0.940	8.6	−0.065	−1.85‐0.602	13.3
BP‐LF, ln mmHg^2^	0.701	0.183–0.889	34.0	0.428	−0.521‐0.786	23.9
BRS‐LF, ln ms/mmHg	0.717	0.236–0.894	15.7	0.407	−0.679‐0.783	16.5
Hemodynamics 40–45 min post						
Stroke volume, mL	0.504	−0.151‐0.803	14.5	0.711	0.243–0.891	16.4
Cardiac output, L/min	0.357	−0.338‐0.731	10.4	0.625	−0.028‐0.861	12.3
SVR, dyn·s·cm^−5^	0.046	−0.718‐0.564	12.2	0.393	−0.715‐0.777	12.2
Blood pressure 55–60 min post						
Sys BP, mmHg	0.926	0.810–0.975	4.2	0.930	0.812–0.974	4.2
Dia BP, mmHg	0.862	0.632–0.948	4.7	0.820	0.510–0.933	5.9
Mean BP, mmHg	0.894	0.718–0.960	3.8	0.894	0.713–0.960	4.5

Abbreviations: BP, blood pressure; BP‐LF, low frequency power of beat‐to‐beat systolic blood pressure; BRS‐LF, baroreflex sensitivity from low frequency band; CI, confident interval; CV%, coefficient of variation; HF, high frequency power of RR intervals; ICC, intraclass correlation coefficient; LF, low frequency power of RR intervals; PWV, pulse wave velocity; RMSSD, root mean square of successive differences between RR inetervals; SVR, systemic vascular resistan.

### 3.1 Case example of raw signals

**FIGURE 1:**
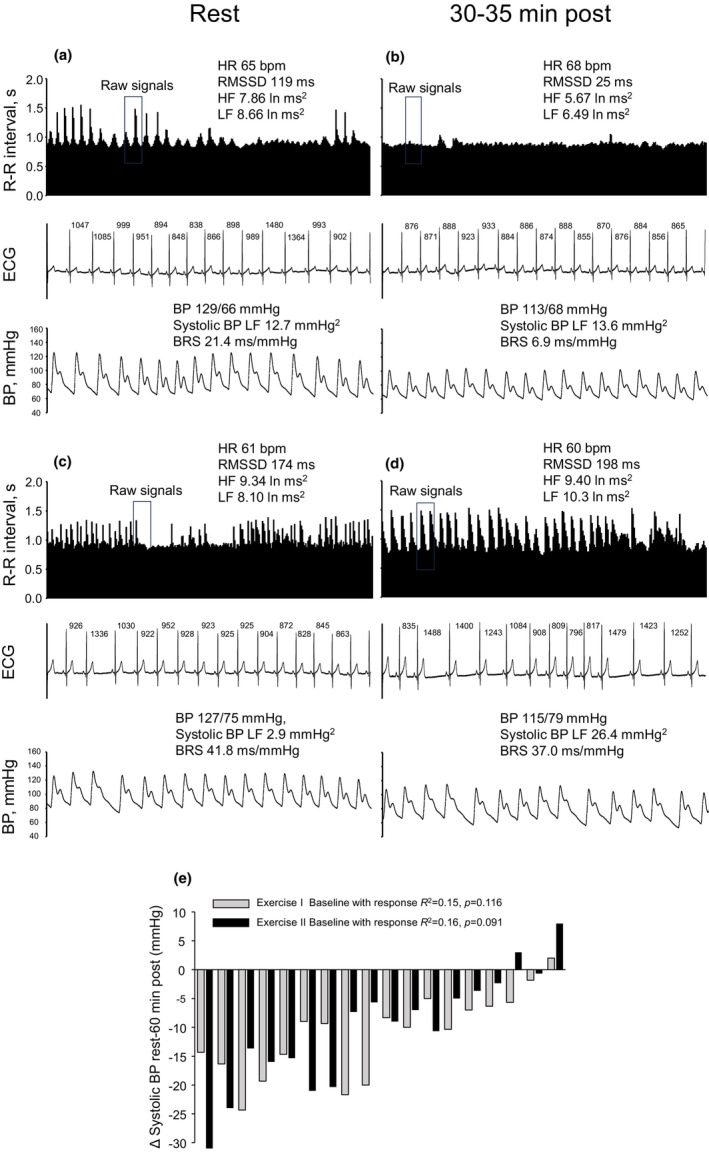
Five min of RR‐intervals tachogram, 15‐s portions of electrocardiogram (ECG) and continuous blood pressure (BP) in a 22‐year‐old male (Vo_2_peak 49 mL/kg/min) at rest (a) and during recovery (b) for the first exercise and corresponding signals at rest (c) and after exercise (d) for the second exercise. Individual changes in systolic BP from rest to 60‐min recovery after both aerobic exercises (e). Spearman's correlation between resting systolic BP and the change from rest to 60‐min recovery. Data were sorted according to average responses.

Figure 1 shows example of cardiovascular signals at rest and after exercises for one subject (22‐year‐old male, Vo_2_max 49 mL/kg/min). The recorded 5‐min RR‐interval tachogram includes abrupt and very large RR‐interval lengthening periods within respiration frequency as evidence of very high cardiac vagal activity. However, both tachograms include tens of seconds lasting fixed‐like RR‐interval dynamics almost without respiratory sinus arrhythmia (Figure 1a,c). Additionally, the LF oscillation of systolic BP is very high at rest one (Figure 1a lower panel) compared to rest two (Figure 1c lower panel), revealing differences in peripheral sympathetic stimulation between these two resting measurements. However, resting BP is at the equal level in both conditions (129/66 vs. 127/75 mmHg) despite differences in autonomic regulation. Cardiac and peripheral autonomic regulation shows very different dynamics between recovery one (Figure 1b) and recovery two (Figure 1d), despite rather equal changes in systolic BP from rest to recovery (−16 mmHg vs. −12 mmHg). RR‐intervals oscillation is more like random dynamics which is not associated with respiration at the last minutes of the tachogram (Figure 1d). Figure 1e shows the individual changes in systolic BP from the baseline to the recovery phase after both aerobic exercises. The change in systolic BP from rest to the 60‐min recovery phase did not correlate with resting systolic BP.

## 4 DISCUSSION

This study's main finding is that the reproducibility of HRV variables analyzed over 5 min is from moderate to excellent at a resting condition before exercise but only from poor to moderate in postexercise condition in young, healthy participants with a wide range of aerobic exercise capacity. Additionally, the reproducibility of systolic BP variability, BRS, CO, SV, and SVR is only from poor to good before and after exercise. The reproducibility of systolic BP, as an outcome of all overlapping regulatory mechanisms, is excellent at rest and after exercise. Furthermore, a clear and statistically significant reduction in systolic BP was observed 60 min after both exercises. However, large inter‐individual differences in postexercise systolic BP responses were obvious in normotensive participants, from −30 mmHg decreased to 8 mmHg increased systolic BP compared to the baseline, which were not correlated with baseline systolic BP. Most importantly, the reproducibility of systolic BP after moderate intensity aerobic exercise is excellent measured 30–60 min during recovery from exercise. This finding may help to design study protocols to investigate the mechanisms of post exercise BP physiology.

### 4.1 Heart rate variability

Previous studies have shown the relative reproducibility to be from moderate to excellent when analyzed by ICC at resting condition (Nunan et al., 2009; Sandercock et al., 2005; Sinnreich et al., 1998). The ICC reflects the ability of a test to differentiate between individuals and is therefore considered a measure of relative test‐to‐test reliability. Additionally, 95% CI for ICC should be reported, because there is a 95% chance that the true ICC value lands on any point between low and high ICC. The statistical evaluation and methods for analyzing reproducibility vary between studies and, for example, many studies do not provide 95% CI for ICC (al Haddad et al., 2011; Gerritsen et al., 2003). We reported ICC and 95% CI according to the latest recommendations by Koo and Li (2016). The present study agrees with previous ones that reproducibility of both vagally mediated HRV indices (RMSSD and HF‐power) and the LF power of RR intervals are from moderate to excellent at rest in a laboratory condition. Measures of the CV may alternatively be considered measures of absolute reliability because they reflect the trial‐to‐trial noise. The present CV values at rest (9%–21% for all HRV indices) agree with previous studies investigating resting HRV indices (Nunan et al., 2009; Sandercock et al., 2005; Sinnreich et al., 1998).

We found no studies regarding the reproducibility of HRV indices beyond 10 min after aerobic acute exercise. We wanted to study the reproducibility of HRV over a longer recovery period when heart rate is back to baseline level. Only the heart rate showed moderate to excellent relative reproducibility in the postexercise condition in this study. The lowest value of 95% CI for ICC lands below 0.10 for all HRV indices and systolic BP variability in postexercise condition. Therefore, the relative reproducibility of these variables measured 30 min after a typical aerobic exercise training at moderate intensity is poor in young participants with a wide range of aerobic capacity.

### 4.2 Baroreflex sensitivity

Dietrich et al. (2010) (Dietrich et al., 2010) used the same spontaneous BRS calculation methods and statistics for children (age 11.2 ± 0.7 years) to analyze reproducibility as in the present study. The relative reproducibility for children in BRS was poor from a resting supine position (ICC 0.49 and 95% CI 0.21–0.70) and improved in a standing position (ICC 0.61 and 95% CI 0.39–0.77). The absolute reliability was 13.8% at rest and 12.6% at standing (Dietrich et al., 2010). The relative and absolute reproducibility in their study agrees with our results in young healthy participants. The relative reproducibility of systolic BP variability and BRS (expressed as 95% CI in ICC) was from poor to moderate before and after exercise measured at seated position in the present study.

Maestri et al. (2009) reported a relative reproducibility of spontaneous BRS in a supine position with the same methods as in the present study in healthy adults (age 38 ± 8 years) (Maestri et al., 2009). Relative reproducibility was clearly better in their study than in our study (ICC 0.71 and 95% CI 0.51–0.83). The reproducibility of BRS in other studies is difficult to compare to the present study due to the different BRS calculation methodologies and statistical interpretations. Taken together, the relative and absolute reproducibility of spontaneous BRS is markedly better in the studies investigated in older adults (>30 years) compared to the present study in young adults (<30 years) (Gao et al., 2005; Gerritsen et al., 2003; Iellamo et al., 1996).

### 4.3 Hemodynamics

The relative reproducibility of systolic BP has been shown to be from good to excellent and diastolic BP from poor to moderate after equal exercise training with our study (Fecchio et al., 2017, 2020). Absolute reproducibility has also been shown to be better for systolic BP than any other hemodynamic or autonomic function variables in both the previous and the present studies (Fecchio et al., 2017, 2020). Interestingly, reproducibility of SV, CO, and SVR in the postexercise condition is poor in both our study and a previous one (Fecchio et al., 2020). BP is regulated via these hemodynamic variables, but the reproducibility is still markedly worse than any BP variables in a postexercise condition. This can be explained by individual differences in BP regulatory mechanisms, particularly in the postexercise condition. It has been estimated that 50% of participants presented decreased BP after exercise due to the reduction of CO, while the other 50% had a decrease in SVR, potentially leading to equal postexercise BP (Forjaz et al., 2004). The change in SV from baseline to recovery was the only variable in the present study associated with the change in systolic BP after exercise but not after the control measurement.

### 4.4 Physiological interpretation

The physiological mechanisms for poor reproducibility after exercise in HRV and BRS variables are unknown. Our data in this study show that beat‐to‐beat RR‐interval dynamics could be markedly different between two postexercise conditions (Figure 1b,d). This could be due to the very high level of vagal outflow to the heart, particularly in young participants with above average aerobic exercise capacity. Most importantly, coactivation of a vagal and sympathetic stimulus to the heart occurs during specific physiological conditions, such as during a high level of circulating norepinephrine, cold face immersion, and after acute exercise (Tulppo et al., 1998;Tulppo et al., 2005; Tulppo et al., 2011). Simultaneous activation of a cardiac vagal and sympathetic stimulus results in abrupt and marked RR interval changes in beat‐to‐beat RR‐interval dynamics or, totally different, fixed RR‐interval dynamics without detectable respiratory sinus arrhythmia (Tulppo et al., 2005; Tulppo et al., 2011). The portions of a very high level of respiratory sinus arrhythmia that suddenly change to the fixed‐like RR‐interval dynamics were observed in the present study as a potential marker of coactivation of vagal and sympathetic outflow to the heart. It is well known that vagal outflow to the heart begins to decline at the age of 30–40 years (Pikkujämsä et al., 1999). These changes in vagal outflow to the heart with aging may potentially explain differences in reproducibility between young and older participants. Extremely high vagal activity in young subjects (estimated by high values of HF power and RMSSD), particularly in postexercise conditions, results in a sudden and marked RR‐interval lengthening or in fixed RR‐interval dynamics that may not be observed in older subjects. These changes in RR‐interval dynamics in a postexercise condition result in a weakening of reproducibility in autonomic function metrics. Also, the potential changes in lifestyle, including use of caffeine and alcohol, stress, diet, physical activity, and hydration status results in differences in daily cardiac and peripheral autonomic regulation.

### 4.5 Limitations

We did not use the “gold standard” method (Co_2_ rebreathing techniques) to measure SV, CO, and SVR in the present study. This was for practical reasons, because the measurement session was already rather busy and time demanding for participants. However, our results are in line with a recent study's before and after exercise measured with the Co_2_ rebreathing techniques (Fecchio et al., 2020).

## 5 CONCLUSION

The reproducibility of HRV variables is from moderate to excellent at resting condition before exercise but only from poor to moderate in a postexercise condition in young participants. The physiological mechanisms for poor reproducibility of these variables in the postexercise condition are due to the very high vagal outflow and the delicate interaction between the vagal and sympathetic regulation of vascular system. The reproducibility of systolic BP is from good to excellent at rest and after aerobic exercise.

## AUTHOR CONTRIBUTIONS

M.P.T., R.I.P.V., and V.P.Y conceived and designed research; M.P.T., R.I.P.V., and V.P.Y performed experiments; M.P.T., R.I.P.V., and V.P.Y analyzed data; M.P.T., R.I.P.V., V.P.Y, and H.V.H. interpreted results of experiments; M.P.T., R.I.P.V., and V.P.Y, drafted manuscript; M.P.T., R.I.P.V., V.P.Y, H.V.H, K.J.J., and R.K. edited and revised manuscript; M.P.T., R.I.P.V., V.P.Y, H.V.H, K.J.J., and R.K. approved final version of manuscript.

## FUNDING INFORMATION

The study was funded by the Finnish Foundation for Cardiovascular Research, Helsinki, Finland (Project numbers 200190, 230117) and Business Finland (project number 41656/2020), Helsinki, Finland.

## CONFLICT OF INTEREST STATEMENT

No conflicts of interest, financial or otherwise, are declared by the authors.

## ETHICS STATEMENT

The study was conducted according to the Declaration of Helsinki and approved by the ethical committee of the Northern Ostrobothnia Hospital District in Oulu, Finland (03/2022). All participants provided written informed consent and had the right to terminate the study at any time.

## Data Availability

The data that support the findings of this article are not publicly available due to privacy and ethical concerns. They can be requested from the corresponding author at mikko.tulppo@oulu.fi.

## References

[phy270007-bib-0001] al Haddad, H. , Laursen, P. B. , Chollet, D. , Ahmaidi, S. , & Buchheit, M. (2011). Reliability of resting and postexercise heart rate measures. International Journal of Sports Medicine, 32, 598–605. 10.1055/S-0031-1275356/ID/20/BIB 21574126

[phy270007-bib-0002] Costa, E. C. , Dantas, T. C. B. , De Farias, L. F. , Frazão, D. T. , Prestes, J. , Moreira, S. R. , Ritti‐Dias, R. M. , Tibana, R. A. , & Duhamel, T. A. (2016). Inter‐ and intra‐individual analysis of post‐exercise hypotension following a single bout of high‐intensity interval exercise and continuous exercise: A pilot study. International Journal of Sports Medicine, 37, 1038–1043. 10.1055/S-0042-112029/ID/R5575-0016/BIB 27676151

[phy270007-bib-0003] Dietrich, A. , Rosmalen, J. G. M. , Althaus, M. , van Roon, A. M. , Mulder, L. J. M. , Minderaa, R. B. , Oldehinkel, A. J. , & Riese, H. (2010). Reproducibility of heart rate variability and baroreflex sensitivity measurements in children. Biological Psychology, 85, 71–78. 10.1016/J.BIOPSYCHO.2010.05.005 20553793

[phy270007-bib-0004] Fecchio, R. Y. , Brito, L. C. , Peçanha, T. , & Forjaz, C. L. M. (2020). Consistency of hemodynamic and autonomic mechanisms underlying post‐exercise hypotension. Journal of Human Hypertension, 35(11), 1003–1011. 10.1038/s41371-020-00452-w 33262435

[phy270007-bib-0005] Fecchio, R. Y. , Chehuen, M. , Brito, L. C. , Peçanha, T. , Queiroz, A. C. C. , & de Moraes Forjaz, C. L. (2017). Reproducibility (reliability and agreement) of post‐exercise hypotension. International Journal of Sports Medicine, 38, 1029–1034. 10.1055/S-0043-118009/ID/R6115-0025 28922683

[phy270007-bib-0006] Forjaz, C. , Cardoso, C. J. , Rezk, C. , Santaella, D. , & Tinucci, T. (2004). Postexercise hypotension and hemodynamics: The role of exercise intensity. The Journal of Sports Medicine and Physical Fitness, 44, 54–62. 15181391

[phy270007-bib-0007] Gao, S. A. , Johansson, M. , Hammarén, A. , Nordberg, M. , & Friberg, P. (2005). Reproducibility of methods for assessing baroreflex sensitivity and temporal QT variability in end‐stage renal disease and healthy subjects. Clinical Autonomic Research, 15, 21–28. 10.1007/S10286-005-0224-4/METRICS 15768198

[phy270007-bib-0008] Gerritsen, J. , TenVoorde, B. J. , Dekker, J. M. , Kingma, R. , Kostense, P. J. , Bouter, L. M. , & Heethaar, R. M. (2003). Measures of cardiovascular autonomic nervous function: Agreement, reproducibility, and reference values in middle age and elderly subjects. Diabetologia, 46, 330–338. 10.1007/S00125-003-1032-9/TABLES/4 12687330

[phy270007-bib-0009] Iellamo, F. , Legramante, J. M. , Raimondi, G. , Castrucci, F. , Massaro, M. , & Peruzzi, G. (1996). Evaluation of reproducibility of spontaneous baroreflex sensitivity at rest and during laboratory tests. Journal of Hypertension, 14, 1099–1104. 10.1097/00004872-199609000-00009 8986910

[phy270007-bib-0010] Kinnunen, H. , Rantanen, A. , Kentt, T. , & Koskimäki, H. (2020). Feasible assessment of recovery and cardiovascular health: Accuracy of nocturnal HR and HRV assessed via ring PPG in comparison to medical grade ECG. Physiological Measurement, 41, 4NT01. 10.1088/1361-6579/AB840A 32217820

[phy270007-bib-0011] Kiviniemi, A. M. , Hautala, A. J. , Karjalainen, J. J. , Piira, O.‐P. , Lepojärvi, S. , Ukkola, O. , Huikuri, H. V. , Tulppo, M. P. , & Hartiala, J. (2015). Acute post‐exercise change in blood pressure and exercise training response in patients with coronary artery disease. Frontiers in Physiology, 5, 526. 25628572 10.3389/fphys.2014.00526 PMC4290526

[phy270007-bib-0012] Koo, T. K. , & Li, M. Y. (2016). Cracking the code: Providing insight into the fundamentals of research and evidence‐based practice a guideline of selecting and reporting Intraclass correlation coefficients for reliability research. Journal of Chiropractic Medicine, 15, 155–163. 10.1016/j.jcm.2016.02.012 27330520 PMC4913118

[phy270007-bib-0013] Lamberts, R. P. , van Erp, T. , Javaloyes, A. , Eken, M. M. , Langerak, N. G. , & Tam, N. (2024). Reliability of recovery heart rate variability measurements as part of the lamberts submaximal cycle test and the relationship with training status in trained to elite cyclists. European Journal of Applied Physiology, 124(6), 1659–1668. 38198009 10.1007/s00421-023-05385-z PMC11130066

[phy270007-bib-0014] Liu, S. , Goodman, J. , Nolan, R. , Lacombe, S. , & Thomas, S. G. (2012). Blood pressure responses to acute and chronic exercise are related in prehypertension. Medicine and Science in Sports and Exercise, 44, 1644–1652. 10.1249/MSS.0B013E31825408FB 22899388

[phy270007-bib-0015] Maestri, R. , Raczak, G. , Torunski, A. , Sukiennik, A. , Kozłowski, D. , La, R. M. T. , & Pinna, G. D. (2009). Day‐by‐day variability of spontaneous baroreflex sensitivity measurements: Implications for their reliability in clinical and research applications. Journal of Hypertension, 27, 806–812. 10.1097/HJH.0B013E328322FE4B 19300111

[phy270007-bib-0016] Nunan, D. , Gay, D. , Jakovljevic, D. G. , Hodges, L. D. , Sandercock, G. R. H. , & Brodie, D. A. (2009). Validity and reliability of short‐term heart‐rate variability from the polar S810. Medicine and Science in Sports and Exercise, 41, 243–250. 10.1249/MSS.0B013E318184A4B1 19092682

[phy270007-bib-0017] Nuuttila, O.‐P. , Korhonen, E. , Laukkanen, J. , & Kyröläinen, H. (2022). Validity of the wrist‐worn polar vantage V2 to measure heart rate and heart rate variability at rest. Sensors, 22(1), 137. 10.3390/s22010137 PMC8747571 35009680

[phy270007-bib-0018] Perkiömäki, N. , Auvinen, J. , Tulppo, M. P. , Ollila, M. M. , Junttila, J. , Perkiömäki, J. , Karhunen, V. , Puukka, K. , Järvelin, M. R. , Huikuri, H. V. , & Kiviniemi, A. M. (2019). Childhood growth patterns and cardiovascular autonomic modulation in midlife: Northern Finland 1966 birth cohort study. International Journal of Obesity, 43(11), 2264–2272. 10.1038/s41366-019-0333-0 30718821

[phy270007-bib-0019] Pikkujämsä, S. M. , Mäkikallio, T. H. , Sourander, L. B. , Räihä, I. J. , Puukka, P. , Skyttä, J. , Peng, C. K. , Goldberger, A. L. , & Huikuri, H. V. (1999). Cardiac interbeat interval dynamics from childhood to senescence. Circulation, 100, 393–399. 10.1161/01.CIR.100.4.393 10421600

[phy270007-bib-0020] Sandercock, G. R. H. , Bromley, P. D. , & Brodie, D. A. (2005). The reliability of short‐term measurements of heart rate variability. International Journal of Cardiology, 103, 238–247. 10.1016/J.IJCARD.2004.09.013 16098384

[phy270007-bib-0021] Seipäjärvi, S. M. , Tuomola, A. , Juurakko, J. , Rottensteiner, M. , Rissanen, A. P. E. , Kurkela, J. L. O. , Kujala, U. M. , Laukkanen, J. A. , & Wikgren, J. (2022). Measuring psychosocial stress with heart rate variability‐based methods in different health and age groups. Physiological Measurement, 43, 055002. 10.1088/1361-6579/AC6B7C 35483348

[phy270007-bib-0022] Sinnreich, R. , Kark, J. D. , Friedlander, Y. , Sapoznikov, D. , & Luria, M. H. (1998). Five minute recordings of heart rate variability for population studies: Repeatability and age–sex characteristics. Heart, 80, 156–162. 10.1136/HRT.80.2.156 9813562 PMC1728778

[phy270007-bib-0023] Springer, M. V. , Malani, P. , Solway, E. , Kirch, M. , Singer, D. C. , Kullgren, J. T. , & Levine, D. A. (2022). Prevalence and frequency of self‐measured blood pressure monitoring in US adults aged 50‐80 years. JAMA Network Open, 5, e2231772. 10.1001/JAMANETWORKOPEN.2022.31772 36103183 PMC9475387

[phy270007-bib-0024] Tulppo, M. P. , Kiviniemi, A. M. , Hautala, A. J. , Kallio, M. , Seppänen, T. , Mäkikallio, T. H. , & Heikki, H. V. (2005). Physiological background of the loss of fractal heart rate dynamics. Circulation, 112, 314–319. 10.1161/CIRCULATIONAHA.104.523712 16009791

[phy270007-bib-0025] Tulppo, M. P. , Kiviniemi, A. M. , Hautala, A. J. , Kallio, M. , Seppänen, T. , Tiinanen, S. , Mäkikallio, T. H. , & Huikuri, H. V. (2011). Sympatho‐vagal interaction in the recovery phase of exercise. Clinical Physiology and Functional Imaging, 31, 272–281. 10.1111/J.1475-097X.2011.01012.X 21672134

[phy270007-bib-0026] Tulppo, M. P. , Mäkikallio, T. H. , Seppänen, T. , Airaksinen, J. K. E. , & Huikuri, H. V. (1998). Heart rate dynamics during accentuated sympathovagal interaction. American Journal of Physiology. Heart and Circulatory Physiology, 274, H810–H816. 10.1152/AJPHEART.1998.274.3.H810/ASSET/IMAGES/LARGE/AHEA4030604.JPEG 9530192

